# Supplementary material for: Sub-wavelength modulation of χ(2) optical nonlinearity in organic thin films
Source: Nat Commun. 2017 Jan 27;8:14269. doi: 10.1038/ncomms14269 (PMC5290150; doi:10.1038/ncomms14269)
Supplement: Supplementary Information — Supplementary Figures, Supplementary Notes and Supplementary References. [file ncomms14269-s1.pdf]

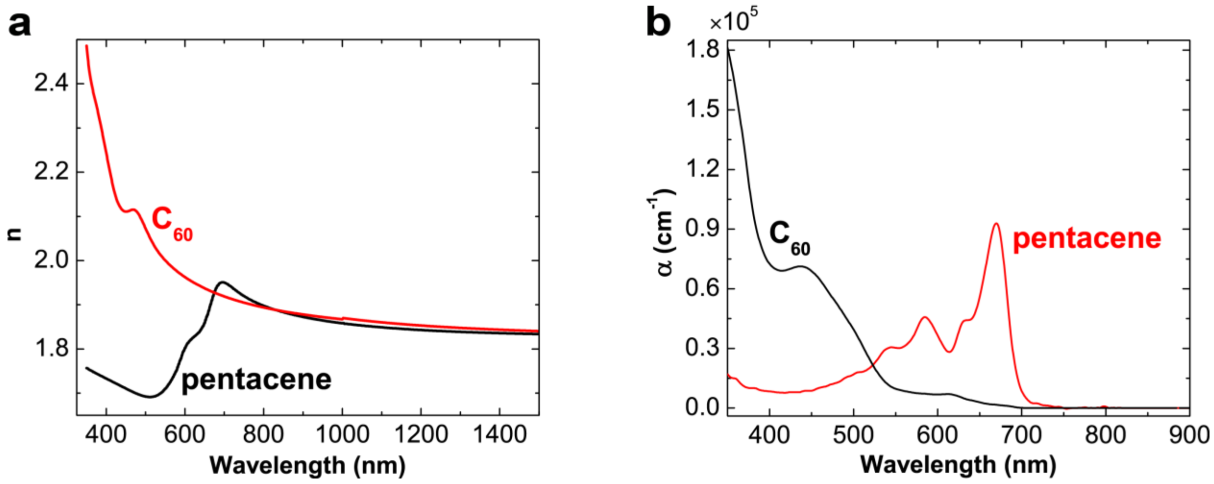

Supplementary Figure 1| Linear optical properties of pentacene and C_{60}. a, Linear refractive indices of pentacene and C_{60}. **b**, Absorption coefficients of pentacene and C_{60}. The linear complex refractive index dispersions are measured via variable angle ($\theta = 55^\circ, 65^\circ, 75^\circ$) spectroscopic ellisometry in the wavelength range $250 < \lambda < 1700$ nm for films deposited on a Si substrate. The refractive index dispersions are fit using a Tauc-Lorentz model, with extinction coefficients independently verified from absorption coefficients (α) measured with a UV/VIS/NIR spectrophotometer according to the relationship, $A = 1 - T - R = 1 - \mathrm{e}^{-\alpha L}$, where L is the film thickness and A, T, and R are the absorptance, transmittance, and reflectance, respectively.

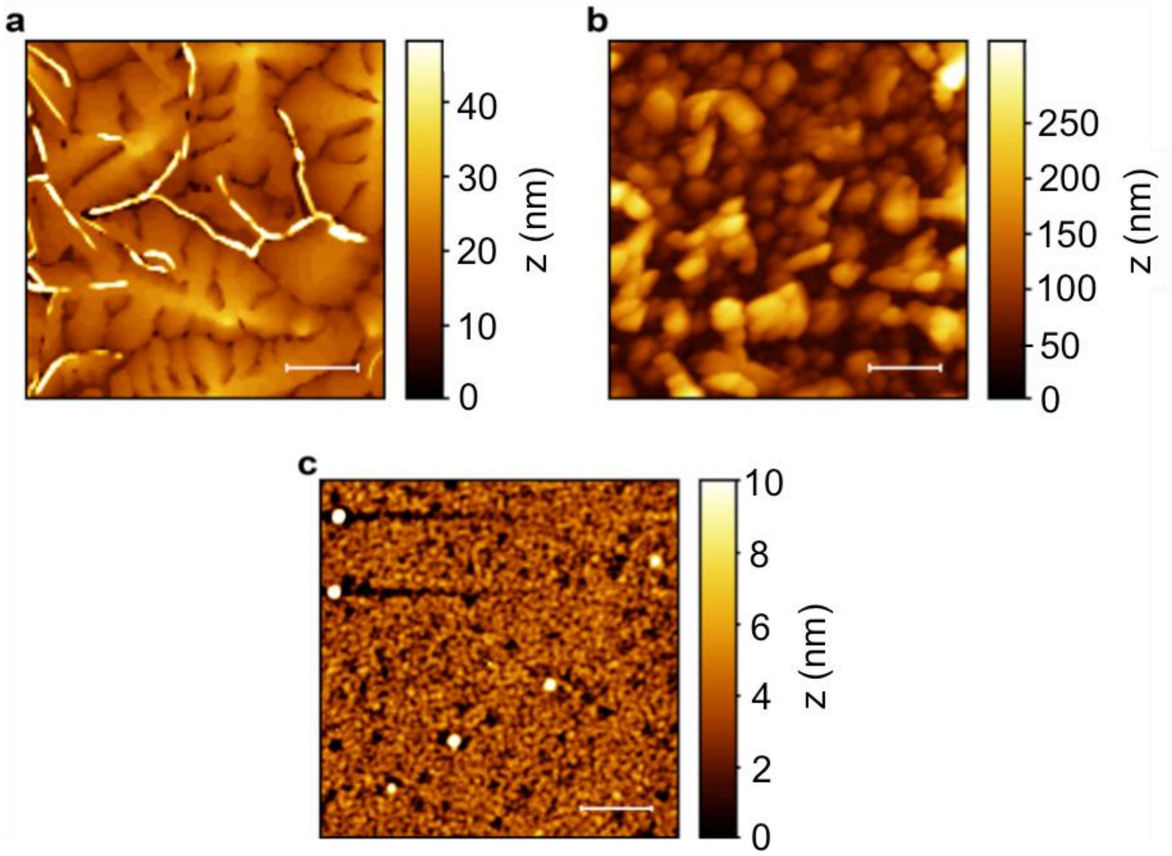

Supplementary Figure 2| Pentacene thin film and DAS multilayer morphology. 5 μm × 5 μm atomic force microscope images of **a**, a single 18 nm pentacene layer; **b**, four DAS repeat units consisting of [pentacene (5 nm)/C_{60} (5 nm)/CBP (5 nm)]; and **c**, fifteen DAS repeat units consisting of [CBP (5 nm)/rubrene (5 nm)/C_{60} (5 nm)]. For both samples in **a** and **b**, pentacene layers are grown at a substrate temperature of 60 ℃ and a rate of 0.3 ~ 0.6 Å s^{-1} on a sapphire substrate. For the sample in **c**, rubrene layers are grown at a substrate temperature of room temperature and a rate of ~1 Å s^{-1}. The scale bar in all of the figures is 1 μm.

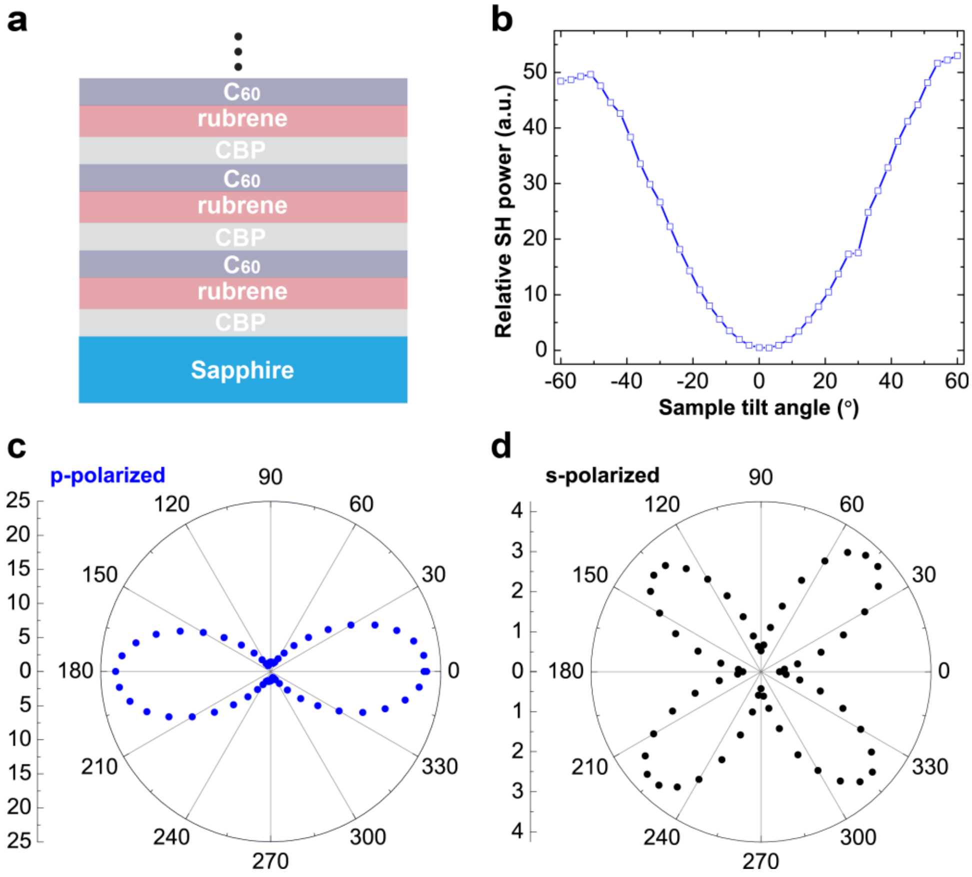

Supplementary Figure 3| SHG from rubrene/C_{60}/CBP DAS multilayers a, Sample structure for rubrene/C_{60}/CBP DAS multilayers grown on a sapphire substrate with individual layer thicknesses, $t_{\mathrm{Rub}} = 5$ nm, $t_{\mathrm{C60}} = 5$ nm, and $t_{\mathrm{CBP}} = 5$ nm. **b**, Tilt scan results for p-polarized SHG measured from a 6 period multilayer. (**c**,**d**) Polar plots showing the dependence of p- and s-polarized SHG power on the pump polarization angle (0° corresponds to p-polarization of the pump). The left-hand axes in **c** and **d** denote the radial scale. The multilayer sample in **a** is grown at a rate of 1 Å s^{-1} at a substrate temperature of 20°C. The SHG tilt scan and polarization patterns shown in **b**, **c**, and **d** are fit using the procedure described in the text to yield a bulk nonlinear coefficient, $d_{33}^{\mathrm{b}} \approx 9$ pm V^{-1} for the layer stack.

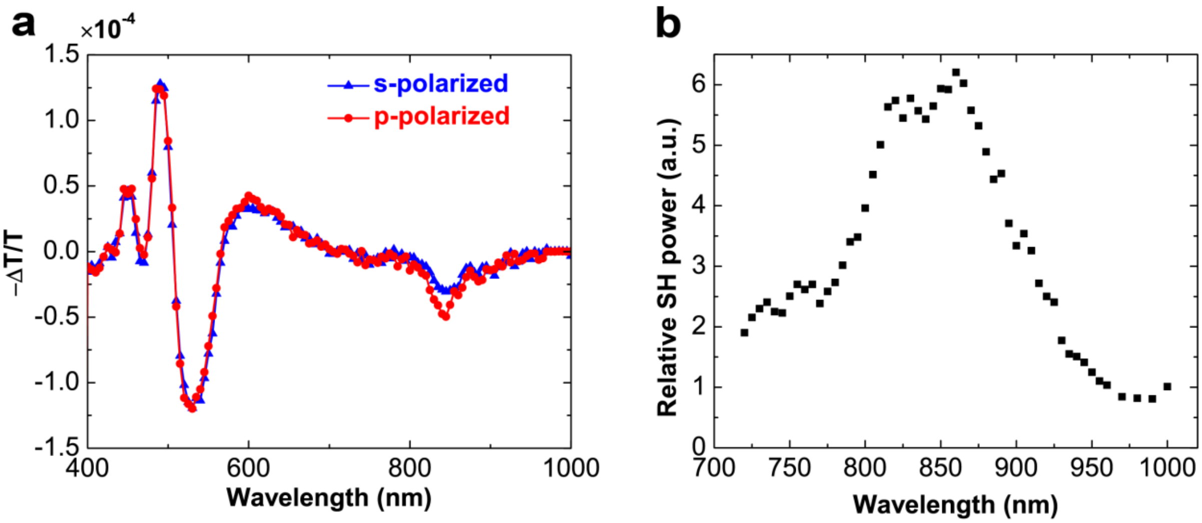

Supplementary Figure 4| Correlation between CT states and $\chi^{(2)}$ nonlinearity. a, Polarized EA spectra of a device with the structure ITO/[rubrene (5 nm)/C_{60} (5 nm)/CBP (5 nm)]×6/Al (80 nm). **b**, SHG excitation spectrum depicting p-polarized SHG power measured as a function of the fundamental wavelength. The data are collected for a 30 period [rubrene/C_{60}/CBP] multilayer using a Spectra-Physics TOPAS-Prime optical parametric amplifier. The EA peak at $\lambda \approx 850$ nm (corresponding to $E_{\mathrm{CT}} = 1.46$ eV) coincides directly with the peak of the SHG resonance as expected from Eqn. 1 in the main text, confirming the strong intermolecular CT contribution to the nonlinear optical response.

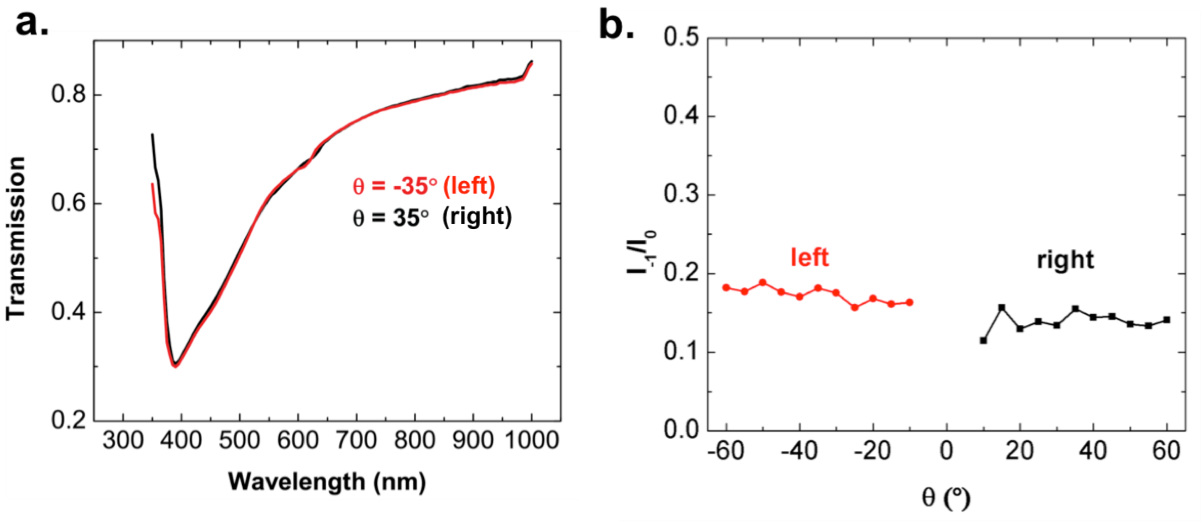

Supplementary Figure 5| Linear oblique angle grating optical characterization. a, White light transmission spectra (i.e. zeroth order diffraction efficiency, T_0) of the oblique-angle deposited DAS grating from Fig. 5b in the main text, measured at similar incidence angles as the 'left' and 'right' nonlinear diffraction measurements. The spectra indicate a negligible blazing effect for T_0 at both the fundamental and second harmonic wavelengths. **b,** Linear diffraction efficiency of the -1 transmitted order (i.e. T_{-1}) for a $\lambda = 405$ nm laser beam close to the second harmonic wavelength as a function of 'left' and 'right' incidence angles (corresponding to negative and positive angles, respectively); it is not possible to measure the diffraction efficiency of the fundamental wavelength since the short grating period does not allow any propagating orders. The slight asymmetry favouring diffraction from left incidence angles is consistent with that observed in Fig. 5b in the main text and confirms a slight blazing effect in the linear T_{-1} diffracted order due to the oblique angle deposition of C_{60}. This effect is qualitatively captured in the numerical grating simulations in Fig. 6 of the main text and stems from the refractive index contrast between C_{60} and the surrounding organic materials at $\lambda \sim 400$ nm.

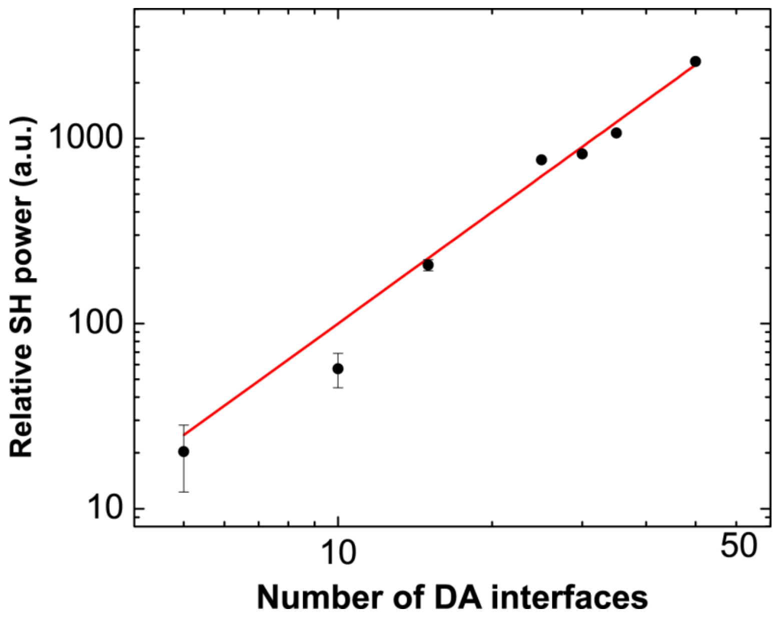

Supplementary Figure 6| Dependence of SH power on the number of rubrene/C_{60}/CBP DAS periods in a series of different samples with individual layer thicknesses maintained at $t_{\mathrm{Rub}} = 5$ nm, $t_{\mathrm{C60}} = 5$ nm, and $t_{\mathrm{CBP}} = 5$ nm. The data exhibit an approximately quadratic dependence (represented by the red line), consistent with a bulk-like nonlinearity originating from the many DA interfaces. Vertical error bars reflect the standard deviation obtained from measuring multiple locations on each sample.

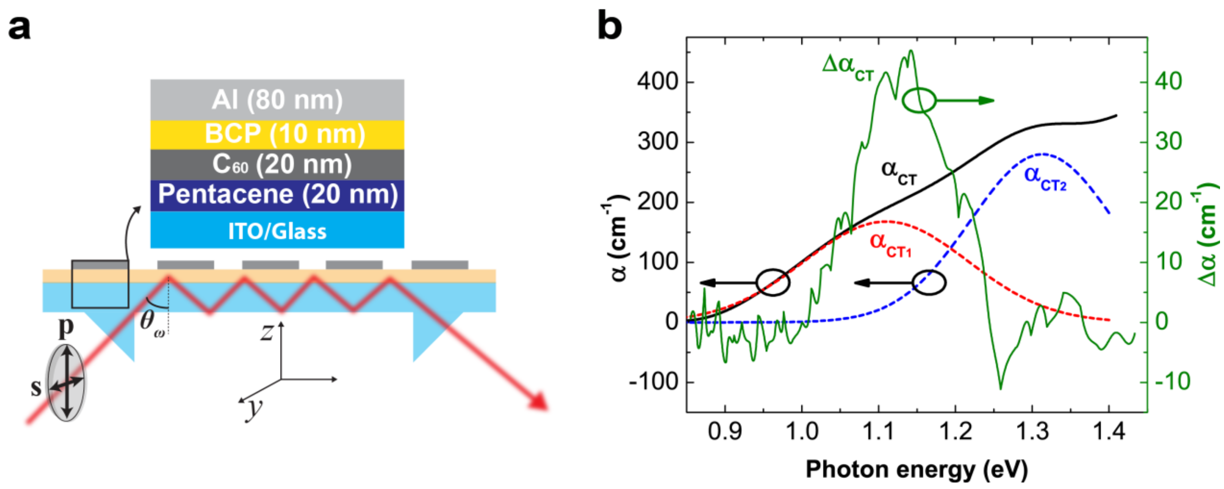

Supplementary Figure 7| Electroabsorption measurements. a, Schematic of the total internal reflection geometry used to increase the signal-to-noise ratio of weak CT state electroabsorption signals. Light is coupled in and out of the device substrate by a pair of right angle prisms and interacts with multiple different devices tied together with a common ITO electrode. **b**, CT state absorption spectrum reproduced from the inset of Fig. 3b of the main text, overlaid on the CT electroabsorption spectrum derived from the difference of the p- and s-polarized EA signals in Fig. 3c to eliminate a non-CT related background that stems from weak electroreflectance of the indium-tin-oxide anode. The lowest energy CT absorption peak (red dashed line) coincides with the positive EA peak, suggesting that the latter stems from an increase in oscillator strength transferred at the expense of nearby states such as the higher energy CT transition (blue dashed line).

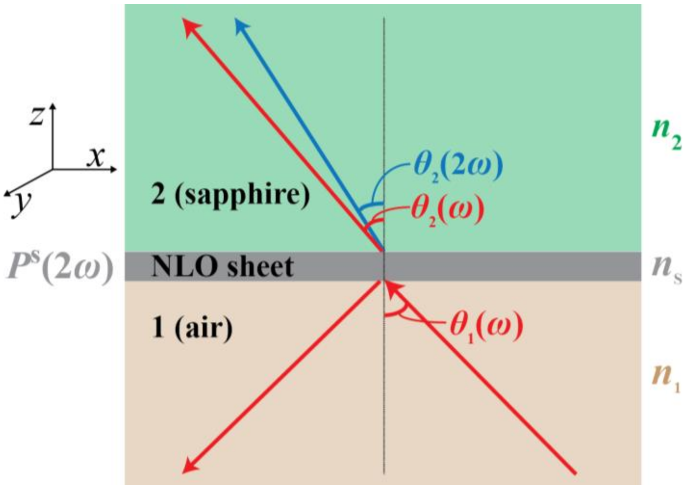

Supplementary Figure 8| Geometry of the nonlinear optical polarization sheet model for transmitted SHG.

Supplementary Note 1| Nonlinear surface polarization model for SHG

Transmitted SHG from the organic thin film samples in Fig. 2 of the manuscript is modelled following the work of Shen[1] in terms of a nonlinear polarization sheet sandwiched between two different linear isotropic media as illustrated in Supplementary Figure 8. In this model, an electric field at the second harmonic frequency (2ω) transmitted to medium 2 is generated from a nonlinear sheet polarization:

$$\boldsymbol{P}^{\boldsymbol{s}}(2\omega) = \boldsymbol{p}^{\boldsymbol{s}}(2\omega)\delta(\mathrm{z})\exp(\mathrm{i}\boldsymbol{k}\cdot\boldsymbol{r} - \mathrm{i}2\omega t)\,, \tag{1}$$

induced by the incoming fundamental field in medium 1.

The nonlinear Maxwell wave equation in turn relates the SH radiation field to P^s according to:

$$\nabla\times(\nabla\times\boldsymbol{E}) + \left(\frac{n_\mathrm{s}^2}{c^2}\right)\left(\frac{\partial^2}{\partial t^2}\right)\boldsymbol{E} = \left(-\frac{4\pi}{c^2}\right)\left(\frac{\partial^2}{\partial t^2}\right)\boldsymbol{P}^{\boldsymbol{s}}. \tag{2}$$

Introducing the electromagnetic boundary conditions between each medium subsequently enables solution for the p- and s-polarized SH fields in medium 2:

$$E_\mathrm{p}(2\omega) = \frac{\mathrm{i}4\pi k_2}{n_1^2(2\omega)k_{2z} + n_2^2(2\omega)k_{1z}}\left[k_{1\mathrm{z}}p_\mathrm{x}^\mathrm{s} - \left(\frac{n_1^2(2\omega)}{n_\mathrm{s}^2(2\omega)}\right)k_\mathrm{x}p_z^\mathrm{s}\right]\exp(\mathrm{i}\boldsymbol{k_2}\cdot\boldsymbol{r} - \mathrm{i}2\omega t), \tag{3}$$

$$E_\mathrm{s}(2\omega) = \frac{\mathrm{i}4\pi k_2}{k_{2z} + k_{1z}}\left[\frac{k_2 p_y^\mathrm{s}}{n_2^2(2\omega)}\right]\exp(\mathrm{i}\boldsymbol{k_2}\cdot\boldsymbol{r} - \mathrm{i}2\omega t), \tag{4}$$

where $\boldsymbol{k_2}$, k_{1z}, k_{2z}, and k_x are the SH wave vector in medium 2, the *z*-component of the SH wave vector in medium 1, the *z*-component of the SH wave vector in medium 2, and the conserved in-plane (*x*-component) SH wave vector, respectively. The linear refractive index of the polarization sheet is n_s, and $p_{x,y,z}^\mathrm{s}$ are the *x*, *y*, and *z* components of the nonlinear polarization. Finally, the linear refractive indices of medium 1 at the fundamental and SH frequencies, and of medium 2 at the SH frequency are $n_1(\omega)$, $n_1(2\omega)$, and $n_2(2\omega)$, respectively.

In the simplified case when medium 1 and medium 2 have the same dielectric properties, there is no boundary and the fields become:

$$E_{\mathrm{p0}}(2\omega) = \frac{\mathrm{i}4\pi\omega}{c}\left(\chi_{\mathrm{ijk}}^{(2),\mathrm{s}} - \frac{k_x(2\omega)}{k_{1z}(2\omega)}\chi_{\mathrm{ijk}}^{(2),\mathrm{s}}\right)E_{1\mathrm{j}}(\omega)E_{1\mathrm{k}}(\omega), \tag{5}$$

$$E_{\mathrm{s0}}(2\omega) = \frac{\mathrm{i}2\pi k_2^2(2\omega)}{n_2^2(2\omega)k_{1z}}\chi_{\mathrm{ijk}}^{(2),\mathrm{s}}E_{1\mathrm{j}}(\omega)E_{1\mathrm{k}}(\omega). \tag{6}$$

The relationship between these simplified fields and those of the full system with a dielectric boundary can be described by the following field correction factors:

$$L_{xx} = \left(\frac{E_{\mathrm{p}}}{E_{\mathrm{p0}}}\right)_x = \frac{2n_2^2(2\omega)k_{1z}}{n_1^2(2\omega)k_{2z} + n_2^2(2\omega)k_{1z}}, \tag{7}$$

$$L_{yy} = \left(\frac{E_{\mathrm{s}}}{E_{\mathrm{s0}}}\right)_y = \frac{2k_{2z}}{k_{2z} + k_{1z}}, \tag{8}$$

$$L_{zz} = \left(\frac{E_{\mathrm{p}}}{E_{\mathrm{p0}}}\right)_z = \frac{2n_2^2(2\omega)k_{2z}(n_1^2(2\omega)/n_{\mathrm{s}}^2(2\omega))}{n_1^2(2\omega)k_{2z} + n_2^2(2\omega)k_{1z}}. \tag{9}$$

Using these field correction factors, the SH fields in Supplementary Equations 3 and 4 can be rewritten as:

$$E_{\mathrm{p}}(2\omega) = \frac{\mathrm{i}2\pi k_2}{n_2^2(2\omega)k_{2z}}(k_{2z}L_{xx}p_x^{\mathrm{s}} - k_x L_{zz}p_z^{\mathrm{s}})\exp(\mathrm{i}\boldsymbol{k_2}\cdot\boldsymbol{r} - \mathrm{i}2\omega t), \tag{10}$$

$$E_{\mathrm{s}}(2\omega) = \frac{\mathrm{i}2\pi k_2}{n_2^2(2\omega)k_{2z}}L_{yy}\,p_y^{\mathrm{s}}\exp(\mathrm{i}\boldsymbol{k_2}\cdot\mathbf{r} - \mathrm{i}2\omega t). \tag{11}$$

Owing to the $C_{\infty v}$ symmetry of the thin film samples under consideration, there are only three nonvanishing components of the susceptibility tensor: $\chi_{15}^{(2),s} = 2d_{15}^{\mathrm{s}}$, $\chi_{31}^{(2),s} = 2d_{31}^{\mathrm{s}}$, and $\chi_{33}^{(2),s} = 2d_{33}^{\mathrm{s}}$. To obtain $p_{x,y,z}^{\mathrm{s}}$ via the relationships $p_{\mathrm{i}}^{\mathrm{s}} = \chi_{\mathrm{ijk}}^{(2),\mathrm{s}}E_{1\mathrm{j}}(\omega)E_{1\mathrm{k}}(\omega)$ and $d_{\mathrm{ijk}}^{\mathrm{s}} = \frac{1}{2}\chi_{\mathrm{ijk}}^{(2),\mathrm{s}}$, the expressions for the total NLO $d_{x,y,z}^{\mathrm{s}}$ coefficients (that represent the NLO response along the *x*, *y*, and *z* sample coordinates) are derived as:

$$d_x^{\mathrm{s}} = d_{15}^{\mathrm{s}} \cos\big(\theta_2(2\omega)\big) \sin\big(2\theta_2(\omega)\big)\, t_{\mathrm{p},1\to\mathrm{s}}^2(\omega) \cos^2\phi, \tag{12}$$

$$d_y^{\mathrm{s}} = 2 d_{15}^{\mathrm{s}} \sin\big(2\theta_2(\omega)\big) t_{\mathrm{p},1\to\mathrm{NLO}}(\omega)\, t_{\mathrm{s},1\to\mathrm{s}}(\omega) \sin\phi \cos\phi, \tag{13}$$

$$\begin{aligned} d_z^{\mathrm{s}} = {} & d_{31}^{\mathrm{s}} \sin\big(\theta_2(2\omega)\big)\, t_{\mathrm{s},1\to\mathrm{s}}^2(\omega) \sin^2\phi + \\ & d_{15}^{\mathrm{s}} \cos^2\big(\theta_2(\omega)\big) \sin\big(\theta_2(2\omega)\big)\, t_{\mathrm{p},1\to\mathrm{s}}^2(\omega) \cos^2\phi + \\ & d_{33}^{\mathrm{s}} \sin^2\big(\theta_2(\omega)\big) \sin\big(\theta_2(2\omega)\big)\, t_{\mathrm{p},1\to\mathrm{s}}^2(\omega) \cos^2\phi\, , \end{aligned} \tag{14}$$

where $\theta_2(\omega)$ and $\theta_2(2\omega)$ are the transmission angles of the fundamental and SH waves in medium 2 determined via Snell's law. The polarization angle of the incident fundamental beam for p- and s-polarizations corresponds to $\phi = 0°$ and $\phi = 90°$, respectively, and $t_{\mathrm{p},1\to\mathrm{s}}(\omega)$ and $t_{\mathrm{s},1\to\mathrm{s}}(\omega)$ are the Fresnel transmission coefficients for p- and s-polarized fundamental waves at the interface between medium 1 and the NLO sheet.

Relating the second harmonic electric field to the intensity via:

$$I(2\omega) = \frac{c n_2(2\omega) |E(2\omega)|^2}{2\pi}\, , \tag{15}$$

then yields, after some manipulation, the absolute p- and s-polarized SH beam powers transmitted into air from medium 2:

$$\begin{aligned} \mathcal{P}_{\mathrm{p}}(2\omega) = {} & \frac{32\pi^3 k_2^2}{A c n_1^2(\omega)\, n_2^4(2\omega) \cos^2\big(\theta_2(2\omega)\big) k_{2z}^2} \times \\ & \Big[(k_{2z}^2 L_{xx}^2 (d_x^{\mathrm{s}})^2 + k_x^2 L_{zz}^2 (d_z^{\mathrm{s}})^2)^2 \big| t_{\mathrm{p},2\to\mathrm{a}}(2\omega) \big|^2 S_{\mathrm{p}}^2(\omega) \Big], \end{aligned} \tag{16}$$

$$\begin{aligned} \mathcal{P}_{\mathrm{s}}(2\omega) = {} & \frac{32\pi^3 k_2^2}{A c n_1^2(\omega)\, n_2^3(2\omega) \cos^2\big(\theta_2(2\omega)\big) k_{2z}^2} \times \\ & \big(L_{yy} d_y\big)^2 \big| t_{\mathrm{s},2\to\mathrm{a}}(2\omega) \big|^2 S_{\mathrm{s}}^2(\omega)\, . \end{aligned} \tag{17}$$

Here, A is the pump spot area, $t_{\mathrm{p},2\to\mathrm{a}}(2\omega)$ is the Fresnel transmission coefficient of the SH wave from medium 2 into air and $\mathcal{P}_{\mathrm{p}}(\omega)$ and $\mathcal{P}_{\mathrm{s}}(\omega)$ are the p- and s- polarized fundamental beam

powers, respectively. In analyzing the Pn/C_{60} bilayers from Fig. 2 of the manuscript, medium 1 is taken to be air, medium 2 is the sapphire substrate, and n_s is the average of Pn and C_{60} refractive indices. We note that, because this model does not account for the fact that both Pn and C_{60} absorb at the second harmonic wavelength, the extracted NLO d-coefficients likely underestimate the actual values.

Supplementary Note 2| Calculation of CT state oscillator strength

The CT state absorption spectrum is deduced from the difference of s- and p-polarized EQE spectra as described previously[2], assuming an effective thickness, $t_{\mathrm{CT}} = 1$ nm, for the CT interfacial absorbing region. Based on this model, the absolute CT state extinction coefficient, k_{CT}, is determined through transfer matrix modelling that incorporates the known optical constants of pentacene and C_{60} given in Supplementary Figure 1. This in turn enables estimation of $\alpha_{\mathrm{CT}} = 4\pi k_{\mathrm{CT}} \lambda^{-1}$ and thus the CT state absorption cross-section, $\sigma_{\mathrm{CT}} = \alpha_{\mathrm{CT}} N_{\mathrm{CT}} t_{\mathrm{CT}}^{-1}$, assuming that the interface CT state density, $N_{\mathrm{CT}} \sim 10^{14}$ cm^{-2}.

Supplementary Note 3| Electroabsorption model for oriented CT states

The energy shift of a localized CT transition due to a static electric field applied normal to the interface (F_z) follows from perturbation theory in terms of the change in dipole moment, $\Delta\boldsymbol{\mu}_{\mathrm{CT}}$, and the change in (tensor-averaged) polarizability, $\langle \Delta\bar{p}_{\mathrm{CT}} \rangle$, between the ground and CT excited states:

$$\Delta E_{\mathrm{CT}} \approx -|\Delta\boldsymbol{\mu}_{\mathrm{CT}}| F_z \cos\theta - \langle \Delta\bar{p}_{\mathrm{CT}} \rangle F_z^2/2 \,, \qquad (18)$$

where θ is the angle between the CT dipole moment and the $\hat{z}$-directed applied electric field, F_z. Taylor expanding the associated change in CT absorption coefficient due to the energy shift:

$$\Delta\alpha_{\mathrm{CT}} = \alpha'_{\mathrm{CT}}\Delta E_{\mathrm{CT}} + \frac{1}{2}\alpha''_{\mathrm{CT}}\Delta E_{\mathrm{CT}}^2\,, \tag{19}$$

and retaining only first harmonic terms from the applied field, $F_z = F_{\mathrm{DC}} + F_{\mathrm{AC}}\cos(\omega t)$, then leads to:

$$\Delta\alpha_{\mathrm{CT},1\omega} = -\alpha'_{\mathrm{CT}}\Delta\mu_{\mathrm{CT}}F_{\mathrm{AC}}\cos\theta - \alpha'_{\mathrm{CT}}\langle\Delta\bar{p}_{\mathrm{CT}}\rangle F_{\mathrm{DC}}F_{\mathrm{AC}} + \alpha''_{\mathrm{CT}}[\Delta\mu_{\mathrm{CT}}^2F_{\mathrm{DC}}F_{\mathrm{AC}}\cos^2\theta]\,, \tag{20}$$

where α'_{CT} and α''_{CT} are the first and second derivatives of the linear CT absorption spectrum with respect to photon energy. The first term on the right-hand side of Supplementary Equation 20 is the linear Stark shift and is only observed when there is a net alignment of CT dipoles (i.e. when $\langle\cos\theta\rangle \neq 0$). In addition, because the applied field mixes the CT state (energy E_{CT}) with other states of similar energy, E_{m} (e.g. other CT states or local D or A exciton states), a transfer of oscillator strength is also possible according to[3]:

$$\Delta\alpha_{\mathrm{CT}} \approx \alpha_{\mathrm{CT}}\frac{\Delta E_{\mathrm{CT}}}{E_{\mathrm{m}} - E_{\mathrm{CT}}}\,. \tag{21}$$

In the case of the Pn/C_{60} EA spectrum shown in Fig. 3c of the main text, we observe that the magnitude of the first harmonic CT EA signal is independent of DC bias, which rules out the latter two terms in Supplementary Equation 20 and confirms that the CT EA arises from the linear Stark effect (in contrast with the higher energy excitonic transitions, which exhibit the usual quadratic Stark effect). Overlaying the field-induced change of CT absorption together with the linear CT absorption in Supplementary Figure 7, it is evident that the EA signal is not described by a derivative lineshape (i.e. arguing against the first term of Supplementary Equation 20) and is instead more consistent with a change in the strength of the lowest energy CT transition highlighted by the red dashed line.

We note that, in the weak microcavity of a solar cell architecture, it is also possible for EA signals to manifest from the electromodulated change in refractive index that accompanies

the absorption changes outlined above due to the Kramers-Kronig relation. A refractive index contribution seems unlikely in this case, however, since the ~1 nm thick CT interfacial region should make any associated phase shift negligible. On the other hand, because at least one additional CT state clearly exists ~0.2 eV above the lowest energy transition (blue dashed line), it seems plausible that a transfer of oscillator strength between the two could dominate the EA spectrum. We therefore tentatively evaluate the magnitude of the EA peak on the basis of Supplementary Equation 21 assuming $E_{\mathrm{m}} - E_{\mathrm{CT}} \approx 0.2$ eV, which in turn leads to an estimate for $\Delta\mu_{\mathrm{CT}} \sim 40$ D. A more detailed analysis of the CT state EA spectrum remains the subject of ongoing work.

SUPPLEMENTARY REFERENCES

1 Shen, Y. Optical second harmonic generation at interfaces. *Annu. Rev. Phys. Chem.* **40**, 327-350 (1989).

2 Brigeman, A. N. *et al.* Revealing the Full Charge Transfer State Absorption Spectrum of Organic Solar Cells. *Adv. Energy. Mater.* DOI: 10.1002/aenm.201601001 (2016).

3 Horvath, A., Weiser, G., Baker, G. & Etemad, S. Influence of disorder on the field-modulated spectra of polydiacetylene films. *Phys. Rev. B* **51**, 2751-2757 (1995).
